# The Impact of Workplace Health Promotion Programs Emphasizing Individual Responsibility on Weight Stigma and Discrimination

**DOI:** 10.3389/fpsyg.2018.02206

**Published:** 2018-11-19

**Authors:** Susanne Täuber, Laetitia B. Mulder, Stuart W. Flint

**Affiliations:** ^1^Department of Human Resource Management and Organizational Behavior, University of Groningen, Groningen, Netherlands; ^2^School of Sport, Leeds Beckett University, Leeds, United Kingdom

**Keywords:** workplace health promotion programs, attribution of controllability, responsibility, weight stigma, weight-based discrimination, obesity

## Abstract

Over time, there has been a steady increase of workplace health promotion programs that aim to promote employees' health and fitness. Previous research has focused on such program's effectiveness, cost-savings, and barriers to engaging in workplace health promotion. The present research focuses on a downside of workplace health promotion programs that to date has not been examined before, namely the possibility that they, due to a focus on individual responsibility for one's health, inadvertently facilitate stigmatization and discrimination of people with overweight in the workplace. Study 1 shows that the presence of workplace health promotion programs is associated with increased attributions of weight controllability. Study 2 experimentally demonstrates that workplace health promotion programs emphasizing individual rather than organizational responsibility elicit weight stigma. Study 3, which was pre-registered, showed that workplace health promotion programs emphasizing individual responsibility induced weight-based discrimination in the context of promotion decisions in the workplace. Moreover, focusing on people with obesity who frequently experience weight stigma and discrimination, Study 3 showed that workplace health promotion programs highlighting individual responsibility induced employees with obesity to feel individually responsible for their health, but at the same time made them perceive weight as *less* controllable. Together, our research identifies workplace health promotion programs as potent catalysts of weight stigma and weight-based discrimination, especially when they emphasize individual responsibility for health outcomes. As such, we offer valuable insights for organizations who aim to design and implement workplace health promotion programs in an inclusive, non-discriminatory way that benefits all employees.

## Introduction

Decades ago, employees worked in environments where smoking was normal and yoga or going for a run during office hours was out of the question. Back then, employers would not have thought about encouraging employees to eat less meat, exercise regularly, and reduce cigarette and alcohol consumption. However, over the course of the last 30 years the interference of employers with their employees' health and lifestyle gained support and is now largely considered appropriate (Goetzel et al., 2014). This is partly due to the aging workforce, which emphasizes the necessity of sustainable employment and partly due to improved insights into the contribution of lifestyle to health outcomes. Consequently, and although the types of intervention and design vary, workplace health promotion programs (WHPP) have become common and accepted (Walters, (n.d.); Mattke et al., 2013). Indeed, the National Institute for Clinical Excellence (2018) state that “All workplaces, particularly large organizations such as the NHS and local authorities should address the prevention and management of obesity, because of the considerable impact on the health of the workforce and associated costs to industry” (p. 3). The present research challenges the assumption that such programs are unanimously beneficial for all parties. Specifically, many WHPP focus on supporting employees to “manage their weight” in response to the current agenda relating to obesity (Public health England, 2018). We propose and test that such framing of responsibility within WHPP forms a potent foundation for weight stigma and discrimination in the workplace. Since experiences of stigma are associated with decreased mental and physical health (e.g., Puhl and Suh, 2015) and with an associated increase in healthcare costs (e.g., Osumili et al., 2016), WHPP might form a cause of what they aim to cure. The current investigation tests the influence of both the presence and focus (individual vs. organizational responsibility) of WHPP on weight stigma and discrimination.

### Workplace health promotion and weight stigma

Healthy employees are the backbone of sustainable employment and productivity (e.g., World Health Report; World Health Organisation, 2002). Given that most people spend two-thirds of their waking hours at work, the workplace represents a logical setting to deliver health and wellbeing interventions (Frase and Gornick, 2012). In alignment, the World Health Organisation (2010) suggested that in the twenty-first century, the workplace should form the primary setting for health promotion. Not surprisingly, therefore, the last thirty years have seen a steady increase in WHPP (Goetzel et al., 2014). According to the 2012 Employer Health Benefits Survey, 94% of large and 63% of small employers offered a WHPP (cf. Chen et al., 2015). While WHPP vary widely in what they target (e.g., disease prevention, employee wellbeing, or lifestyle and health education; Chen et al., 2015), the expectation is that they will benefit employers as well as employees. An area of particular focus for WHPP currently is employees' weight status, and in particular the reduction of weight and the increase of physical activity (e.g., Quintiliani et al., 2007; Schröer et al., 2013). This focus is in response to the high and increasing prevalence of overweight and obesity and associated non-communicable disease across the world (World Health Organisation, 2017), and an appreciation of the influence of workplace issues such as sedentary behavior, prolonged sitting time, and unhealthy food and drink consumption (Schröer et al., 2013). This is reflected in the WHO's Global Plan of Action on Worker's Health 2008-2017 as cited by Quintiliani et al. (2007), pp. 7–8: “Health promotion and prevention of non-communicable diseases should be further stimulated in the workplace, in particular by advocating healthy diet and physical activity among workers, and promoting mental health at work…”

Whilst there are benefits to WHPP, we propose that the current focus on weight that emphasizes employees' responsibility can inadvertently elicit weight stigma. Weight stigma refers to negative attitudes toward a person because of their weight status. People with overweight or obesity are negatively stereotyped as being weak willed, lazy, unintelligent and gluttonous (Puhl and Brownell, 2001; Phelan et al., 2014). Indeed, although a link between prejudice and discrimination is not always evident, negative attitudes toward people with overweight and obesity have been associated with biased treatment (O'Brien et al., 2013). Weight-based discrimination has been reported across a range of settings and among people of all ages and backgrounds. For instance, Phelan et al. (2015) reviewed empirical evidence for obesity stigma in health care settings, noting that many health care providers hold strongly negative stereotypes about people with obesity. Aligning with this observation, weight stigma and discrimination have also been reported in settings that are critical for the prevention and treatment of obesity such as exercise (Schvey et al., 2017), and healthcare facilities (Raves et al., 2016). Relatedly, Tomiyama et al. (2015) found that, while implicit weight bias decreased, explicit weight bias increased between 2001 and 2013 among scientific researchers specializing in obesity and other obesity-related professionals.

Negative stereotypes about people with obesity also lead to discrimination in the workplace. For instance, suitability judgements of applicants in the hiring process or employees for promotion are lower for applicants with obesity (e.g., Flint et al., 2016), people with overweight or obesity, on average, earn less and are more often unemployed (Kim and von dem Knesebeck, 2018). Further, it has been shown that people with obesity are perceived as possessing less leadership qualities compared to normal weight counterparts (O'Brien et al., 2008; Flint and Snook, 2014). In addition, research has reported that employees with obesity have lower starting salaries, are assessed as being less qualified, and work longer hours than normal weight employees (Baum and Ford, 2004; Han et al., 2011). Not surprisingly, experiences of weight stigma and discrimination also may have serious adverse effects on mental health, including compromised psychosocial wellbeing, social isolation, healthcare avoidance, binge eating, body related shame and guilt, and weight gain and development of obesity (e.g., Puhl and Suh, 2015; Mensinger et al., 2018), which can contribute to sickness absence.

The above suggests that, while health promotion in the workplace is considered a prime tool in supporting employee health (e.g., World Health Organisation, 2010), the workplace also is a prime setting where weight stigma and discrimination is experienced (e.g., Roehling et al., 2007; Bartels and Nordstrom, 2013). With regards to WHPP, systematic reviews examining their impact on overweight and obesity (Anderson et al., 2009) and on increasing physical activity (Malik et al., 2014), report modest improvements or inconclusive results. Building on these insights and testing the idea that workplace health promotion can be a mixed blessing, we propose that WHPP affect employees' attributions of how controllable weight is, thereby laying the foundation for weight stigma and discrimination. We expect this effect to be particularly pronounced for WHPP focusing on individual rather than organizational responsibility for health outcomes. Given that weight discrimination is a stressful experience with a host of negative outcomes on psychological and physical health (e.g., Quintiliani et al., 2007; Phelan et al., 2014, 2015), and even increasing mortality risk (Sutin et al., 2015), understanding the impact of WHPP on weight stigma and discrimination is important. In the sections below, we provide greater elaboration for our reasoning on the associations between WHPP, controllability and responsibility attributions, and weight stigma.

### WHPP, controllability and responsibility attributions, and weight stigma

The design and implementation of WHPP has implications for who is held responsible for employee health. Scholars note a shift in focus from occupational health protection to occupational health promotion, which involves an associated change in responsibility from employers to employees (Macdonald and Sanati, 2010). According to Attribution Theory (Weiner, 1985), how people respond to the negative outcomes of others depends predominantly on the (assumed) controllability of the outcome. A large body of research demonstrates that perceived controllability leads to greater blaming of people for negative outcomes (Weiner et al., 1988; Crandall and Martinez, 1996; Weiner, 2000). This is due to a strong link between perceived controllability and ascribed responsibility for negative outcomes (Mantler et al., 2003). This basic link has been shown for various outcomes such as blindness, heart disease, unemployment, AIDS, alcoholism, divorce and obesity (Weiner et al., 1988; Weiner, 1995).

While many studies show that people blame and stigmatize others held responsible for a negative outcome (Crandall and Martinez, 1996; Rudolph and Tscharaktschiew, 2014), recent research on beliefs about the changeability of attributes suggests a more complex relationship. Specifically, labeling obesity as a biologically driven disease on the one hand decreased anti-fat prejudice through decreasing blame (Burnette et al., 2017; Hoyt et al., 2017), but on the other hand, increased anti-fat prejudice through suggesting that people with obesity have an unchangeable essence. The associations between controllability, ascribed responsibility, weight-stigma and weight-based discrimination are thus complex and inconsistent. Understanding these associations is crucial as they directly affect public health policy and the design of health messages (Burnette et al., 2017; Hoyt et al., 2017). Our research contributes to the literature by examining these associations in the context of WHPP.

Indeed, scholars have warned that an ethical consideration highly relevant for WHPP is the risk of blaming the target (van Berkel et al., 2014). This risk is particularly salient regarding lifestyle-related topics and is thought to arise from the focus of most WHPP on the individual employee rather than on the nature of work and the organization itself (van Berkel et al., 2014, p. 2). Van Berkel et al. further concluded that WHPP would contribute to greater individualization of organizational problems, thereby eroding solidarity. Based on interviews with different stakeholder groups about occupational health, van Berkel et al. (2014) found that stakeholders differed in their view of risk factors in occupational health. Whilst employees and occupational physicians considered the job and working conditions as primary risk factors, employers considered employees' lifestyle decisions to be primary risk factors. Hence, employees construe health as the organization's responsibility, while employers construe health as the individual employee's responsibility. These differences align with the notion that, different from prevention, in health promotion the responsibility for health is more ambiguous (Macdonald and Sanati, 2010). However, because WHPP are generally set-up by employers rather than by employees, they will often be based on the perception that occupational health is largely the responsibility of employees (Meershoek et al., 2010).

This focus on employee responsibility can be manifest in the type of program organizations implement but also in the way a WHPP is communicated. Regarding the type of program, WHPP may contain policies that encourage employees to engage in healthy behaviors. For example, providing education about healthy choices regarding food and drinks or providing access to sport facilities. As such, the responsibility of organizations to provide a working environment that inherently evokes health and healthy behaviors (e.g., making the canteen 100% healthy, giving the staircase a more prominent place than the elevator), may be overlooked. With regard to the way the organization communicates a program, a WHPP may be framed in terms of employee or organizational responsibility. For example, organizations may communicate that healthy food in the canteen is meant to encourage employees to make healthy choices (which taps into employee responsibility of making healthy choices). By contrast, they could also communicate that healthy food offered in the canteen prevents employees from being seduced into unhealthy eating (which taps into the organization's responsibility of creating a health-promoting environment).

Overview articles reviewing WHPP that aimed at weight reduction specifically indeed list the above factors as parts of the reviewed programs (e.g., Schröer et al., 2013). Importantly, these authors also note that “The evaluated interventions were implemented at individual, organizational or combined level with a majority of interventions that were individually focused” (p. 9). We argue that a focus on employee responsibility will contribute to the belief that obesity is controllable. As controllability beliefs are associated with higher weight stigma (e.g., Teachman et al., 2003; Flint et al., 2017), such a focus can be expected to result in stigmatization and discrimination toward people with overweight or obesity (e.g., Crandall and Martinez, 1996; Crandall et al., 2001; Mantler et al., 2003).

### The present research

Aiming to test the effects of WHPP on controllability, weight stigma and discrimination, we conducted three studies. As an initial test, Study 1 examined the impact of WHPP Presence on controllability attributions for weight. Study 2 extended the first study's findings by experimentally varying WHPP Presence and WHPP Focus (emphasizing individual vs. organizational responsibility) and examining their impact on weight stigma. Study 3 (pre-registered) further extended this to weight-based discrimination in the context of promotion decisions, and the impact of WHPP Focus on weight bias internalization among employees with overweight and obesity in particular, thereby shedding light on the potential targets' perspective.

All three studies presented in this article were carried out in accordance with the recommendations of the Ethical Commission of the Behavioral Research Lab of the Faculty of Economics and Business (University of Groningen) with written informed consent from all subjects. All subjects gave written informed consent in accordance with the Declaration of Helsinki.

## Study 1

As a first test of our ideas, we conducted a survey, measuring the presence of a WHPP in the organization that people are employed and controllability perceptions of overweight. We hypothesized that weight is perceived as more controllable when a WHPP is present as opposed to when it is absent (Hypothesis 1).

### Methods

#### Participants and procedure

After we instructed M-Turk to recruit 250 respondents, 255 M-Turk workers completed an online survey. Of these, 38% were from non-western countries. Considering that WHPP are more specific for Western countries and that, in non-western countries, due to a lower prevalence of overweight, there is likely a lower focus on weight in public discourse and WHPP, we decided to include respondents from North-America and Western Europe only. Respondents first answered control questions regarding their employment and size of their organization. We excluded 11% who did not work in an organization (but were self-employed or unemployed). This left a sample of 131 respondents (57 female; *M*_*age*_ = 35.2, *SD*_*age*_ = 9.24), which gave us a power of 0.95 for detecting medium effects of *r* = 0.30 in a one-sample correlation (for the size of our main finding, a correlation of 0.20, the power was 0.64). First, the presence of a WHPP was measured and control questions were asked about respondents' involvement with the WHPP and its implementation. Then, perceived controllability of a range of life events, among which health-related events, was measured.

#### Measurements

To assess the *presence of a health program*, the following question was asked, “At the organization where you work, is there a health program installed?” Possible answers were “*yes*,” “*no*,” and “*I don't know*.” The answers “no” and “I don't know” were collapsed, so that the value “1” stood for “WHPP present” (*N* = 63) and “0” for “not aware of a WHPP being present” (*N* = 68).

To assess *controllability of health*, respondents were presented with several life events and for each event were asked to indicate to what extent they thought it was under people's control or determined outside of a person's control. Answers were given in percentages, with 100% representing an event perceived to be completely within a person's control, and 0% representing an event perceived to be completely outside a person's control. In total, four health-related events were presented: “becoming overweight,” “being overweight,” “getting cancer,” and “getting a burnout.” Controllability of becoming overweight and being overweight were highly correlated (*r* = 0.81) and were therefore combined into one index representing the controllability of overweight. The health-related events were embedded in 12 filler events unrelated to health, such as “being unemployed,” “having children,” and “winning an Olympic medal.” We included these filler items to examine whether the presence of a WHPP affects controllability attributions in general or affects controllability attributions of health-related outcomes specifically. The non-health related filler items were combined into one scale as a measure of controllability of non-health related life events (α = 0.79). The events were presented in a randomized order.

### Results

Table 1 provides descriptive statistics and correlations. WHPP presence was only significantly associated with perceptions of the controllability of overweight indicating that respondents who reported that a WHPP was present in their organization perceived overweight as more controllable (*r* = 0.20, *p* = 0.02, Cohen's *d* = 0.41). By contrast, WHPP presence was only marginally associated with perceived controllability of cancer (*r* = 0.15, *p* = 0.09, Cohen's *d* = 0.30) and of non-health events (*r* = 0.16, *p* = 0.06, Cohen's *d* = 0.32). It was unrelated to perceived controllability of burnout.

**Table 1:** Descriptive statistics and correlations for Study 1.

		***M (SD)***	**1**	**2**	**3**	**4**	**5**	**6**	**7**	**8**	**9**	**10**
Control variables	1. Gender (1 = female)	0.44 (0.50)	1.00								
	2. Age	35.2 (9.24)	−0.03	1.00							
	3. BMI		−0.15	0.27**	1.00						
	4. Size organization (9-point scale)	5.95 (2.16)	−0.12	0.16^+^	0.08	1.00					
	5. Own use of Health Program	2.31 (1.29)	0.12	0.01	−0.12	0.06	1.00				
	6. Involvement in implementing HP	1.97 (1.30)	0.01	−0.22**	−0.20	−0.14	0.67**	1.00			
IV	7. Health program present (1 = yes)	0.48 (0.50)	0.05	0.08	0.03	0.25**	0.53**	0.30**	1.00		
DV's	8. Controllability overweight*	73.1 (22.6)	0.08	0.09	−0.11	0.02	0.06	−0.03	0.20*	1.00	
	9. Controllability burn-out*	57.8 (26.1)	0.10	0.14	0.01	−0.02	0.08	0.06	0.02	0.16	1.00
	10. Controllability cancer*	24.3 (23.1)	0.04	−0.09	−0.01	0.12	0.17*	0.27**	0.15^+^	0.10	0.17^+^	1.00
	11. Controllability non-health events*	56.7 (15.0)	0.08	−0.15^+^	−0.11	0.01	0.28**	0.31**	0.16^+^	0.48**	0.43**	0.32**

* In percent (%).

* *p < 0.05*,

** *p < 0.01*.

Upfront we had determined that, to test whether WHPP presence influenced controllability perceptions, we needed to control for alternative variables that could explain this relation, namely those variables that correlated both with WHPP presence and controllability perceptions. As shown by Table 1, WHPP presence was correlated with organization size, own use of WHPP and involvement in implementing the WHPP. While these variables could not explain the relation between WHPP presence and perceived controllability of overweight (as they did not correlate with perceptions of the controllability of overweight), they could explain the marginal relations between WHPP presence on the one hand and controllability perceptions of cancer and controllability perceptions of non-health events on the other hand. To test whether this was the case, we performed a univariate ANCOVA with the presence of a WHPP program as independent variable, perceptions of the controllability of cancer as the dependent variable, and own use and involvement in implementation as covariates. This showed no effect of presence of WHPP program, *F*_(1, 129)_ = 0.99, *p* = 0.32, Cohen's *d* = 0.18; only the effect of involvement in the WHPP implementation (the more involved, the more cancer was perceived as controllable) was significant, *F*_(1, 129)_ = 5.85, *p* = 0.02, Cohen's *d* = 0.43. An equivalent ANCOVA was performed on controllability perceptions of non-health events, revealing no effect of health program *F*_(1, 127)_ = 0.23, *p* = 0.63, Cohen's *d* = 0.002. Again, only the effect of involvement in the HP implementation (the more involved, the more non-health event were perceived as controllable) was significant, *F*_(1, 127)_ = 4.33, *p* = 0.04, Cohen's *d* = 0.37. Thus, the additional analyses demonstrated that the marginal effects of WHPP presence on controllability perceptions of cancer and non-health related life events disappeared when controlling for involvement in implementation and own use of the WHPP.

Overall, these results suggest that WHPP presence is only related to perceived controllability of *overweight* and that this cannot be explained by other variables as measured in this study,

### Discussion

Findings show that the mere presence of a WHPP in organizations was associated with employees' perceptions that overweight is more controllable. This association was not evident for burnout, and the marginally significant relation between presence of a WHPP and controllability of cancer and non-health related events was fully explained by employees' own involvement in implementing a health program. Thus, the association between WHPP presence and health-related events was unique for weight. This supports Hypothesis 1 that weight is perceived as more controllable when a WHPP is present compared to when a WHPP is absent. Findings further suggest that the effect of WHPP presence is less pronounced for other health outcomes. One might speculate whether this effect is due to the visibility of a health outcome which has appeared to affect attributions of controllability and responsibility (e.g., Weiner et al., 1988). It may also be the case that, in line with our reasoning, most WHPP entail policies that are overweight-relevant and incorporate activities that tap into the controllability of weight. In sum, Study 1 provides initial evidence for the proposition that the mere presence of a WHPP affects employees' perceptions of the controllability of weight.

## Study 2

Whilst the correlational patterns in Study 1 support the hypothesis that weight is perceived as more controllable when a WHPP is present compared to when it is absent, the relationship between WHPP and weight stigma was not examined. In addition, causal relationships were not established. In Study 2, we again included a measure of controllability perceptions. However, we specifically aimed to investigate the proposed causal effects of WHPP presence on *weight stigma*. We hypothesized that weight stigma would be higher when a WHPP is present than when a WHPP is absent (Hypothesis 2). Based on our reasoning that weight stigma results mostly from a WHPP emphasizing individual responsibility, we also tested the difference between a WHPP emphasizing individual vs. organizational responsibility. We hypothesized that weight stigma would be higher when a WHPP emphasizes individual as opposed to organizational responsibility for employees' health (Hypothesis 3).

### Methods

#### Participants and design

We ran this experiment in the lab of a European university among undergraduate business students who participated in exchange for course credits. We aimed to reach 120 respondents, but used the end of the scheduled period as a the stopping rule. Ninety-six students (34 female; *M*_*age*_ = 20.4, *SD*_*age*_ = 2.28) participated. Participants were randomly assigned to one of four conditions, namely No WHPP (*N* = 25), WHPP without responsibility information (*N* = 24), WHPP emphasizing organizational responsibility (*N* = 23), and WHPP emphasizing individual responsibility (*N* = 24). The first two conditions are thus comparable with Study 1, as they represent the presence of WHPP (no vs. yes), while the last two conditions allow comparison of WHPP emphasizing either individual or organizational responsibility.

#### Procedure

First, participants answered questions about their gender, age, weight and height (which were later used to calculate their BMI by dividing people's weight in kilos by their height in meters, squared). Then, participants were presented with a declaration about health, ostensibly from their university. Participants were informed that their university was in the process of further developing this declaration, and wanted to present it to various stakeholders, including students. They were further told that the university would like to hear their opinion about it. The hypothetical declaration served to manipulate the presence and the focus of a WHPP. Students read the declaration and were asked to provide their opinion about it in an open question. Afterwards, they were asked to engage in a task supposedly unrelated to the health declaration they just read. Specifically, they read about a new study examining how people perceive each other, before completing a “picture task,” which served as the measure of weight stigma. Participants' then completed questions relating to the controllability of overweight.

#### Manipulation of health program

The Appendix provides a detailed overview of the manipulations used in this research. In all conditions, the declaration stated, “*The University deems it important that employees and students are healthy, have good condition and are not overweight*.” In the no WHPP condition, only this declaration was provided. In the three conditions where a WHPP was present, participants also read that the university would implement several policies to promote the health of employees and students. Policies that could credibly be implemented were chosen with both a focus on individual and organizational responsibility. These were adapting the building to make the stairs more prominent and the elevator a less prominent, provide healthy food in the canteen, and provide more sports facilities. In the WHPP condition without responsibility information, this was all that respondents read. In the WHPP conditions with information about responsibility, the policies were explained in more detail. This differed between the WHPP emphasizing individual responsibility and the WHPP emphasizing organization responsibility with regard to emphasizing how the policies were a matter of effort of the individual employee or student, or of the organization. For example, when explaining adaptions to the building, in the WHPP emphasizing individual responsibility condition, participants read, “*In this way, people will be motivated to take the stairs instead of the elevator*.” In the WHPP emphasizing organization responsibility condition, participants read, “*In this way, taking the stairs becomes the more “logical option” and people automatically will be more inclined to take the stairs instead of the elevator*.” Likewise, there was a difference in the declaration conclusion, where participants read, “*As such, the university appeals to their employees and students to take responsibility for fostering their own health*” when the WHPP emphasized individual responsibility and “*As such, the university takes her responsibility to foster the health of their employees and students*” when the WHPP emphasized organization responsibility.

#### Measures

##### Weight stigma

Weight stigma was measured by a picture task in which the items of the shortened version of the Fat Phobia scale (Bacon et al., 2001) were used. Participants were shown pictures of two persons of which they were asked to imagine that these people were their lecturers. The picture on the left was of a woman with overweight and the picture on the right was of a woman without overweight. The pictures were drawn from a “before-after” image on the internet of the same person (see the Appendix for the pictures that are anonymized for the purpose of this paper). A pilot test had shown that the woman on the left was indeed perceived as overweight and the woman on the right was not. In addition, the woman with overweight was perceived to be friendlier, less attractive, and younger, although these effects were smaller, and some at the advantage of the woman with overweight. The two women did not differ in perceived competence and dressing style (see Appendix for pilot details).

Participants were asked to imagine that these women were their lecturers and were presented with “several attributes that a university lecturer could possess.” For each attribute, participants were asked to indicate whether this attribute fitted the woman on the left or the woman on the right the most. This was done on a 7-point Likert scale (1 = only applicable to the woman on the left, 4 = equally applicable to both, 7 = only applicable to the woman on the right). A number of attributes were presented, among which were 14 Fat Phobia items: industrious, will power, attractive, slow, endurance, active, weak, self-indulgent, likes food, insecure, high self-esteem, well-shapely, overeats, good self-control. As a lower score reflected a stronger association of the attribute with the woman with overweight, the negative items (slow, weak, self-indulgent, likes food, insecure, overeats) were reverse coded. After these 14 items were averaged into one scale (α = 0.84), a high score on this measure reflected weight stigma. Another item, namely “capable as a teacher,” was included to test whether weight stigma would manifest itself in biased perceptions specific for the context of students evaluating teachers.

##### Controllability attributions

Two questions assessed respondents' beliefs about the controllability of weight (“People have little influence on their weight” and “Overweight is something that people cannot change themselves”). These items were reverse coded and combined into a single index of controllability (*r* = 0.37, *p* < 0.001), with higher values indicating greater perceived weight controllability.

##### BMI

For exploratory reasons, participants were asked to fill in their height and weight and, from this, BMI was calculated. Mean BMI was 22.26 kg/m^2^ (*SD* = 2.42), with a minimum of 18.01 kg/m^2^ and a maximum of 30.19 kg/m^2^. Of the respondents, 87.5% had no overweight (BMI < 25 kg/m^2^), 11.5% had overweight (BMI between 25 and 29.9 kg/m^2^), and 1% had obesity (BMI ≥ 30 kg/m^2^).

### Results

#### Analytic strategy

We performed one-way ANOVA's with *post-hoc* LSD tests to compare all four conditions. Power analyses for this analysis showed a power of 0.50 to detect a medium effect size. Results of these analyses are presented in Table 2 and explained in more detail in the following paragraph. To disentangle the effect of WHPP presence (investigated in Study 1) and the focus of the WHPP, and to make it easier to explore with more power whether the effects of these factors were moderated by BMI, we computed two additional factors from the experimental conditions, namely *WHPP Presence* and *WHPP Focus*. For *WHPP Presence*, a dichotomous variable was constructed contrasting the no WHPP condition with the other three conditions collapsed, thereby corresponding to the absence (0) vs. presence (1) of a WHPP. For *WHPP Focus*, the WHPP emphasizing individual responsibility condition was coded as “1,” the no WHPP condition and the WHPP conditions without responsibility information were coded as “0,” and the WHPP organizational responsibility was coded as “−1.” Thus, a higher score reflected an emphasis on individual responsibility. Correlations of these two factors with other variables are shown in Table 3. Power analysis for correlations showed a power of 0.86 to find a medium effect size of *r* = 0.30.

**Table 2:** Means and standard deviations per experimental condition for Study 2.

**Condition**	**Weight stigma**	**Overweight bias—capable as teacher**	**Controllability perceptions**
No WHPP	5.16^a^ (0.54)	4.28^b^ (0.98)	5.46^b^ (1.30)
WHPP—no responsibility information	5.27^ab^ (0.58)	4.63^b^ (0.97)	6.04^a^ (0.88)
WHPP—organization responsibility	5.10^a^ (0.72)	4.48^b^ (0.99)	6.00^ab^ (1.12)
WHPP—individual responsibility	5.58^b^ (0.68)	5.21^a^ (1.02)	6.21^a^ (0.67)

*Within columns, subscripts that share a letter do not differ significantly*.

**Table 3:** Descriptive statistics and correlations for Study 2.

		***M (SD)***	**1**	**2**	**3**	**4**	**5**	**6**	**7**
Control variables	1. Gender^a^	1.35 (0.48)						
	2. Age	20.40 (2.48)	0.06					
	3. BMI	22.26 (2.42)	−0.15	0.37**				
IVs	4. WHPP Presence^b^	0.74 (0.44)	0.09	−0.18	0.03			
	5. WHPP Focus^c^	0.01 (0.70)	−0.20	0.06	0.03	0.01		
DVs	6. Weight stigma	5.30 (0.61)	0.10	−0.08	−0.12	0.08	0.27**	
	7. Bias—Capability as a teacher	4.65 (1.04)	−0.08	−0.15	−0.11	0.21*	0.25*	0.40**
	8. Controllability	5.92 (1.05)	0.00	0.09	−0.03	0.26*	0.07	0.25*	0.40**

a *1 = male, 2 = female*.

b *1 = yes (N = 71), 0 = no (N = 25)*.

c 1 = individual (N = 24), 0 = no WHPP/ WHPP no information (N = 25 and N = 24, respectively), −1 = organizational (N = 23).

* *p < 0.05*,

** *p < 0.01*.

#### Weight stigma

A one-way ANOVA was performed to test the influence of the different conditions on weight stigma. This showed an overall marginally significant effect of condition, *F*_(3, 92)_ = 2.45, *p* = 0.069, η^2^ = 0.07. LSD *post-hoc* analyses showed that weight stigma was significantly higher in the WHPP individual responsibility condition (*M* = 5.58, *SD* = 0.68), compared to the WHPP organization responsibility condition (*M* = 5.10, *SD* = 0.72) (*p* = 0.015, Cohen's *d* = 0.69) and compared to the no WHPP program condition (*M* = 5.16, *SD* = 0.54), (*p* = 0.032, Cohen's *d* = 0.68). The condition in which the WHPP contained no responsibility information (*M* = 5.27, *SD* = 0.58) did not differ from the other conditions, all *p's* > 0.11 and all Cohen's *d* < 0.50. Table 3 further shows that weight stigma was affected by the focus of the WHPP (*r* = 0.27, *p* < 0.001, Cohen's *d* = 0.56) and not by the mere presence of a WHPP (*r* = 0.08, Cohen's *d* = 0.16, *n.s*.). Together, these results suggest that the presence of a WHPP does not necessarily contribute to weight stigma; however, when WHPP emphasize individual responsibility this does contribute to weight stigma.

#### Impact of BMI

**Figure 1:**
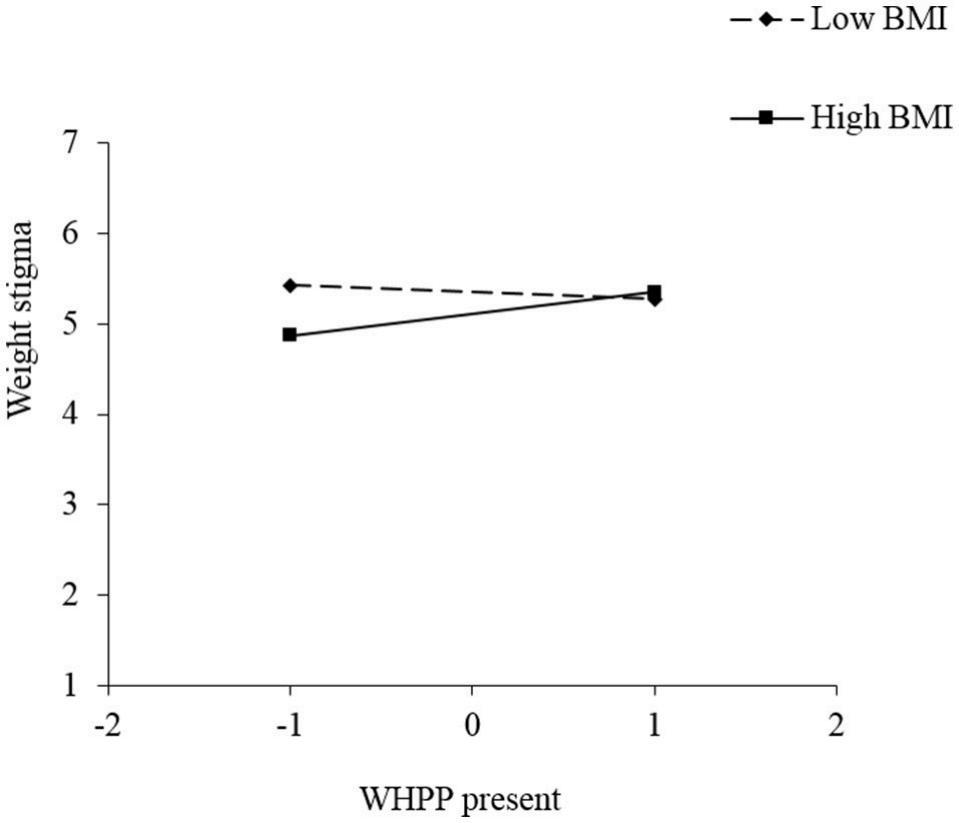
Simple slopes for the effect of *WHPP Presence* on weight stigma, moderated by respondents' BMI, Study 3.

**Figure 2:**
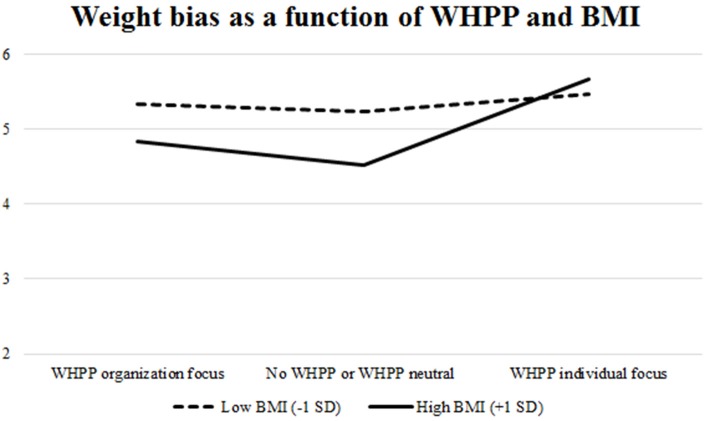
Simple slopes for the effect of *WHPP Focus* on weight stigma, moderated by respondents' BMI, Study 3.

We also explored whether the effect of *WHPP Presence* or *WHPP Focus* on weight stigma depends on someone's own BMI. We performed a regression analysis with weight stigma as the dependent variable and WHPP presence, BMI (standardized) and the interaction term as independent variables. This model was significant, *F*_(3, 95)_ = 2.77, *p* = 0.046 and rendered a marginal main effect of BMI, β = −0.19, *p* = 0.07, Cohen's *d* = 0.39, and a significant BMI × WHPP presence interaction, β = 0.25, *p* = 0.02, Cohen's *d* = 0.50. This interaction is plotted in Figure 1. Simple slopes analyses showed that weight stigma increased due to *WHPP Presence*, but only in participants with a relatively high BMI (β = 0.33, *p* = 0.02, Cohen's *d* = 0.51) and not participants with a relatively low BMI (β = −0.11, *p* = 0.43, Cohen's *d* = 0.16). More specifically, the shape of the figure shows that, in absence of a WHPP, weight stigma is lower among people with a high BMI than among those with a low BMI, and that the presence of a WHPP increase the weight stigma up to the same level as the low BMI participants' weight stigma. We performed a similar regression for *WHPP Focus*. This rendered a significant overall model, *F*_(3, 95)_ = 3.96, *p* = 0.01, a marginal main effect of BMI, β = −0.16, *p* = 0.099, Cohen's *d* = 0.35, and a marginally significant BMI × WHPP focus interaction, β = 0.19, *p* = 0.06, Cohen's *d* = 0.40. This interaction is plotted in Figure 2. Simple slopes analyses showed that weight stigma was affected by *WHPP Focus*, but only in participants with a relatively high BMI (β = 0.47, *p* = 0.002, Cohen's *d* = 0.68) and not participants with a relatively low BMI (β = 0.06, *p* = 0.67, Cohen's *d* = 0.09). Hence, when the focus does *not* lie on individual responsibility, people with a high BMI experience lower levels of weight stigma than people with a low BMI. But an individual focused WHPP increased the level of weight stigma up to the same level of the low BMI participants' weight stigma.

The results suggest that participants reported more weight stigma when exposed to WHPP emphasizing individual responsibility. Also, people with a higher BMI reported more weight stigma both when a WHPP was present and when exposed to WHPP emphasizing individual responsibility compared to people with a lower BMI.

#### Other weight-biased perceptions

We tested the influence of the experimental conditions on students' work-related biased perceptions in the context of teaching. Hence, we performed a one-way ANOVA to examine the item “capable as teacher” as dependent variable. There was an overall significant effect, *F*_(3, 92)_ = 3.94, *p* = 0.01, Cohen's *d* = 0.70. Means and *post-hoc* differences are presented in Table 2 (Cohen's d of the largest significant difference was 0.93 and Cohen's d of the smallest significant difference was 0.58). These results indicate that the WHPP emphasizing individual responsibility increased work-context related biased perceptions toward a person with overweight, compared to all other conditions. Table 3 shows that the bias regarding capability as a teacher was affected by both the presence (*r* = 0.21, Cohen's *d* = 0.43) and the focus of the WHPP (*r* = 0.25, Cohen's *d* = 0.52). This result suggests that implementing a WHPP, especially when it emphasizes individual responsibility, induces people to regard people with overweight as less suitable for a specific job.

We also investigated whether the influence of WHPP presence and focus on biased perceptions of capability as a lecturer depended on respondents' own BMI. This was not the case, as the regression analyses did not show any (marginally) significant interactions between BMI and WHPP (presence or focus).

#### Controllability

A one-way ANOVA was performed to test the influence of the experimental conditions on attributions of the controllability of weight. This showed an overall marginally significant effect of condition, *F*_(3, 92)_ = 2.48, *p* = 0.066, Cohen's *d* = 0.59. LSD *post-hoc* analyses showed that respondents perceived overweight, compared to the condition without a WHPP, to be more controllable in the WHPP-no responsibility information condition (Cohen's *d* = 0.52) and in the WHPP emphasizing individual responsibility condition (Cohen's *d* = 0.72). WHPP Focus did not affect perceived controllability of weight. This aligns with Table 3, which shows that attributions of the controllability of weight were significantly influenced by the WHPP Presence (*r* = 0.26, Cohen's *d* = 0.54) rather than by WHPP Focus.

### Discussion

Study 2 varied the presence of a WHPP and its focus on individual or organizational responsibility for health in a higher education institution. In line with the findings of Study 1, the mere presence of a WHPP led to higher perceived controllability of weight. While controllability perceptions were not influenced by the WHPP's focus on individual or organization responsibility, in line with predictions, WHPP focus (and not so much the mere presence of WHPP) *did* affect weight stigma. Respondents displayed significantly more weight stigma when the WHPP emphasized individual responsibility compared to organizational responsibility for health. WHPP emphasizing individual responsibility also elicited a more particular work-related bias against people with overweight, namely with regard to capability as a lecturer.

Thus, consistent with our reasoning, to the extent that workplace health promotion emphasizes individual rather than organizational responsibility for employee health, weight stigma is evoked. Arguably, stigmatizing thoughts and biases about people with overweight is not the same as discriminatory behaviors toward people with overweight. However, in line with previous research (e.g., O'Brien et al., 2008; Flint et al., 2016), it is expected that biases about people with overweight lead to discriminatory behavior. After all, there is ample evidence that both conscious and unconscious biases about certain groups lead to discriminatory behavior toward members of these groups (e.g., Phelan et al., 2014). To test this, Study 3 aimed to examine the effects of WHPP Focus on weight-based discrimination in the context of a promotion decision. This study tests the expectation that people show greater weight discrimination when exposed to WHPP that endorse individual compared to organizational responsibility for health (Hypothesis 4).

Another interesting finding of Study 2 was that, while, in absence of a WHPP, people with a high BMI showed less weight bias than people with a low BMI, the WHPP changed this. The presence of a WHPP, especially the WHPP that emphasizes individual responsibility, increased the weight stigma only among people with a high BMI up to the same level as people with a low BMI. This finding suggests that the perspective of potential targets of weight stigma in the workplace warrants greater attention. WHPP that emphasize individual responsibility, might lead to people with a high BMI perceiving that they are to be blamed for their weight, thereby inducing self-stigmatization or weight bias internalization (Durso and Latner, 2008). Therefore, in Study 3 we aimed to explore the target's perspective. Thus far, we have focused on those making the judgments; however, in Study 3 we also test the effect of WHPP on weight bias internalization amongst people with overweight or obesity. Specifically, Study 3 tests the prediction that people report more weight bias internalization after exposure to a WHPP emphasizing individual compared to organizational responsibility (Hypothesis 5a) and that this effect is more pronounced in people with a high rather than low BMI (Hypothesis 5b). Note that the statistical power of Study 2 is rather low, and results should therefore be interpreted with caution. We sought to recruit more respondents to reach adequate statistical power in Study 3. To achieve sufficient variance in respondents' weight-status, that would allow testing the effects of WHPP Focus on employees with overweight and obesity, we aimed for a large sample of US citizens.

## Study 3

### Methods

Study 3 was pre-registered. A link to the complete pre-registration of the hypothesis and procedures with regard to sampling, stopping rule and data-analysis can be viewed at https://osf.io/69qmc/?view_only=None by clicking “view registration form.” All procedures as described in the method and result section are in accordance with this pre-registration.

#### Participants and design

The experiment was posted on MTurk as a study about “Decision Making in HR” and people who worked in HR were especially encouraged to participate. Payment was $3 plus a chance to win a $10 bonus. Two hundred and fifty-one MTurk users who were employed and located in the US participated in the study. They were randomly assigned to the conditions of a 2 (WHPP Focus: individual vs. organizational) between-subjects factor by 2 (Candidate Weight-Status: normal weight vs. overweight) within-subjects factor design. Thirteen respondents (5.2%) did not seriously engage with the writing task that was part of manipulating WHPP Focus and were removed from further analyses. This left a sample of 238 respondents (52% male; *M*_*age*_ = 35.76, *SD*_*age*_ = 10.15), of whom 21% worked in HR, either currently or in the past. Seventy-one (71) percent had at least some experience with hiring people (varying from “a little” to “a great deal”). Amongst the respondents, 10% reported high school as their highest level of education, 23% “some college,” 12% a 2-year college degree (Associates), 42% a 4-year college degree (BA, BS), 10% a master's degree, and 3% a doctoral or a professional degree.

#### Procedure

All materials for this study, including the manipulations and measures, can be found in the Appendix. Respondents were asked to take the role of the HR manager in “Sturdation”, a big construction company. In this role, their first task was to write a statement about a WHPP that Sturdation was planning to implement. This task and the preparation for the task served to manipulate WHPP Focus. In preparing respondents for the writing task, they were informed that Sturdation decided to implement a WHPP. After introducing the content of the program and Sturdation's viewpoint on who is responsible for employee health (individual vs. organization), a manipulation check question was asked. The envisioned WHPP and Sturdation's viewpoint on health were then repeated and respondents were asked to write a persuasive text for the advisory board of Sturdation. They were instructed to make the viewpoint of Sturdation clear, convince the supervisory board of this viewpoint, and explain how the measures being implemented in the WHPP align with Sturdation's viewpoint. Respondents could not continue with the survey if they wrote a text shorter than 180 characters. This was not part of the instructions, and respondents only became aware of this if they tried to continue with a text of less than 180 words. After writing this text, respondents were asked three questions about their agreement with the WHPP and their satisfaction with Sturdation. These questions were intended to let the manipulation sink in and were not part of hypotheses testing.

In the second task, which served as the dependent measure of weight-based discrimination, respondents took an advisory role in an internal application procedure for the vacancy of senior policy advisor with advising on international branding as main responsibility. They saw a short CV and photo of two candidates named Lucy and Megan (see Attachment)^1^. For half of the respondents, a photo of a women with overweight was coupled with Lucy's CV and a photo of a woman with normal weight was coupled with Megan's CV. For the other half of the respondents, this was the other way around. We used the same photos as in the weight stigmatization task in Study 2. The order that the candidates were presented, as well as whether Lucy or Megan was with overweight, was randomized. Respondents indicated the hireability of each candidate. A suspicion probe was presented that asked respondents to write down what they thought the research question was for this study. Then, respondents completed measures of weight bias internalization, two measures of perceived controllability of weight, and a second manipulation check. Finally, demographic questions were asked, amongst which was weight and height.

#### Manipulation WHPP

Depending on the experimental condition, respondents read different information about the WHPP and Sturdation's viewpoint on employee health. In the text below, the individual responsibility instruction is in brackets and the organizational responsibility instruction is in Italics:

“The Workplace Health Promotion Program is based on the viewpoint that the health of an employee is the responsibility of *the organization* (each individual employee). This is because the health of a person is very much influenced by *the environment he/she lives and works in, in terms of availability of healthy food and opportunities to exercise* (his/her own behavior in terms of eating an exercise). Therefore, the task of Sturdation is to *offer a healthy work environment* (encourage employees to take their responsibility).”

The WHPP's content in both conditions concerned four actions, namely healthy food in the canteen, taking the stairs rather than the elevator, offering a health check, and influencing employees' movement in the office while at work. The implementation of these actions differed between conditions. In the individual responsibility condition, the actions were aimed at encouraging employees to behave in healthy ways, while the actions in the organizational responsibility condition were aimed at adapting the working environment so that it evoked healthy behavior amongst employees. In addition, the action of offering a health check differed between conditions where, in the individual responsibility condition, follow-up actions on the health check were at the cost of the employee, whilst in the organization responsibility condition, follow-up actions were covered by the organization. The actions as described in the two different conditions are presented in the Appendix.

#### Measurements

##### BMI

BMI was calculated in the same way as in Study 2. For two respondents this rendered a missing value, as there was doubt about the unit they used to fill in their height or weight. Mean BMI was 27.19 kg/m^2^ (*SD* = 6.18), with a minimum of 16.65 kg/m^2^ and a maximum of 53.16 kg/m^2^. Of the respondents, 40.3% were without overweight (BMI < 25 kg/m^2^), 33.4% with overweight (BMI between 25 and 29.9 kg/m^2^), and 26.4% with obesity (BMI ≥ 30 kg/m^2^).

##### Manipulation checks

We checked the WHPP manipulation with two questions. Specifically, respondents were asked, “According to the viewpoint of Sturdation, who is responsible for the health of individual employees?” directly after the WHPP manipulation, and “In your own opinion, who is responsible for the health of individual employees?” after the measure of Weight Bias Internalization. Both questions were answered on a 7-point Likert scale (1 = the employee is solely responsible, 7 = the organization is solely responsible). These questions were reverse coded, such that higher values reflect greater perceived individual responsibility for health. In addition to these questions, the text that respondents wrote about Sturdation's vision on the WHPP was coded with regard to whether they wrote about individual responsibility, organizational responsibility, or mixed/unclear. This coding was performed by a researcher who was blind to experimental conditions.

##### Weight bias internalization

The 11-item Modified Weight Bias Internalization Scale (Pearl and Puhl, 2014) was used. An example item is “Because of my weight, I feel that I am just as competent as anyone” (reverse coded). Answers were given on a 7-point Likert scale (1 = strongly disagree, 7 = strongly agree). The scale was highly reliable (α = 0.95).

##### Controllability of overweight

Two measures were employed. First, the slider measure in Study 1 was used, but now focusing on the overweight items (which made all other items distraction items). Since “becoming overweight” and “being overweight” rendered similar results in Study 1, we now only included “being overweight.” The second measure was the 8-item Beliefs About Obese Persons Scale (BAOP; Allison et al., 1991). An example item of this scale is “Obesity is usually caused by overeating.” Answers were given on a 6-point Likert scale (1 = I strongly disagree, 6 = I strongly agree). Reliability was insufficient (α = 0.61), but reliability increased to a satisfactory level (α = 0.79) after removal of the first and last item. Items were coded such that for both measures, higher scores reflected greater perceived controllability.

##### Weight-based discrimination

Weight-based discrimination was operationalized by comparing hireability judgments of the two candidates. Lower hireability judgement for the candidate with overweight compared to the candidate without overweight indicated weight-based discrimination. For both candidates, hireability judgements were measured with four items relating to the candidates' skills and competences (α = 0.91 and α = 0.92 for the normal weight and the candidate with overweight, respectively). The Appendix provides the detailed measurements.

##### Suspicion

Answers that respondents gave to the question asking them what they thought the research question was for this study were coded on suspicion. More specific, an independent coder, blind for the condition to which participants were assigned, coded for each answer whether it showed that the participant thought that the study was about weight bias in evaluating job candidates (0 = no, 1 = yes). Answers that were coded as suspicious were, for example, “Not sure. Possibly trying to gain insight into weight biases” and “I think this research aims at observing whether participants will hire the woman without overweight or the woman with overweight, once they have been exposed to health concerns in the workplace.”

### Results

The coding of the suspicion probe made clear that a significant portion of our respondents issued suspicion about the hypothesis (69 respondents, 27.5%). Consistent with our pre-registered analytical strategy, for the analysis of Hypothesis 4 (about weight-based discrimination), we removed the respondents who indicated suspicion about the hypotheses. However, the number of suspicious respondents was larger than we had imagined upfront. Naturally, removing so many respondents reduces our statistical power. Therefore, we additionally report the (non-pre-registered) analysis including these respondents. For Hypothesis 5 (about weight-bias internalization), we continued testing our hypotheses on weight bias internalization including the 69 suspicious respondents. Our reason for doing this was not only that including these respondents increases power, but also that the suspicion about the hiring task was less relevant for measuring weight bias internalization than for measuring weight-based discrimination. This is something we had not realized during the pre-registration. To abstain from p-hacking, we did not do any further analyses without the 69 suspicious respondents.

For all our hypothesis-testing and exploratory analyses, we tested for studentized residual outliers and determined the cut-off point using a Bonferroni correction. In the following, we report outliers when they were detected and explain how we dealt with them (which was in line with the pre-registration).

Analyses supplementing the analyses reported below are provided in the Appendix.

#### Manipulation checks

Of all respondents, 95% wrote a text that matched the experimental condition they were assigned to; 1.7% wrote a text contrary to their condition; 3.4% wrote a mixed or unclear text. As we could not conclude for sure that respondents providing texts contrary to their condition or mixed or unclear texts did not keep to the instructions (as it was not forbidden to use opposite or irrelevant arguments), we chose not to exclude these respondents from the analysis, in line with our pre-registration. Further, we regressed WHPP Focus (individual responsibility = 1, organizational responsibility = −1), BMI (standardized) and their interaction term on the two manipulation check questions concerning responsibility attributions. Both models were significant, *F*_(3, 228)_ = 247.67, *p* < 0.001, *R*^2^ = 0.77 and *F*_(3, 228)_ = 19.30, *p* < 0.001, *R*^2^ = 0.20, for Sturdation's and respondents' own viewpoint, respectively. Importantly, for both questions only a main effect of condition was evident (Sturdation: β = 0.0.87, *t* = 27.05, *p* < 0.001; Own opinion: β = 0.45, *t* = 7.57, *p* < 0.001). Thus, a WHPP emphasizing individual responsibility elicited significantly greater attributions of individual responsibility, as opposed to organizational responsibility for health. The manipulation can thus be considered successful.

#### Hypothesis testing: weight-based discrimination

Hypothesis 4 stated that weight-based discrimination would be greater when the WHPP emphasized individual as opposed to organizational responsibility. A mixed-model ANOVA with WHPP Focus as between-subjects factor and candidate weight-status as within-subjects factor was performed by including hireability judgements of the candidate with overweight and without overweight as the dependent variable. Power analyses for this analysis using G^*^Power (Faul et al., 2007) showed a power of 0.76 and of 0.91 to detect a medium effect size for the analysis with and without the exclusion of suspicious respondents, respectively.

##### With exclusion of suspicious respondents (as preregistered)

We identified four outliers that did not significantly affect the regression coefficient of interest, which is why we did not remove them. A main effect was found of candidates' weight-status, *F*_(1, 163)_ = 4.62, *p* = 0.03, Cohen's *d* = 0.33. The candidate was judged as less hireable when she was with overweight (*M* = 5.76, *SD* = 1.17) compared to without overweight (*M* = 5.90, *SD* = 0.96), indicating weight-based discrimination. The interaction between WHPP Focus and Candidate Weight-Status did not reach significance, *F*_(1, 167)_ = 1.99, *p* = 0.16, Cohen's *d* = 0.012. Nevertheless, given the a priori prediction we performed tests for simple main effects. These showed that weight-based discrimination was evident only when the WHPP emphasized individual responsibility, *F*_(1, 167)_ = 5.69, *p* = 0.02, Cohen's *d* = 0.37, but not when the WHPP emphasized organizational responsibility, *F*_(1, 167)_ = 0.31, *p* = 0.58, Cohen's *d* = 0.09.

##### With inclusion of suspicious respondents (not pre-registered)

We performed the same analysis including the 69 suspicious respondents. Six outliers were detected that did not affect the regression coefficients, and were therefore not removed. Again a main effect of the candidates' weight-status was observed, *F*_(1, 236)_ = 11.14, *p* = 0.001, Cohen's *d* = 0.43 (candidate with overweight candidate: *M* = 5.80, *SD* = 1.11; candidate without overweight: *M* = 6.00, *SD* = 0.91). This effect was qualified by a significant WHPP Focus × Candidate Weight-Status interaction, *F*_(1, 236)_ = 4.27, *p* = 0.04, η^2^ = 0.02, Cohen's *d* = 0.27. Tests for simple main effects showed that weight-based discrimination was evident only when the WHPP emphasized individual responsibility, *F*_(1, 236)_ = 14.85, *p* < 0.001, Cohen's *d* = 0.50, but not when the WHPP emphasized organizational responsibility, *F*_(1, 236)_ = 0.74, *p* = 0.374, Cohen's *d* = 0.11.

##### Conclusions hypothesis 4

Results of the two analyses reported above thus support Hypothesis 4. Nevertheless, we wish to frame this conclusion with some care as the analyses that were done according to our pre-registered analyses (thus excluding the 69 suspicious participants) partly supported Hypotheses 4: whereas the simple main effects were in line with the hypothesis, the interaction was not significant (*p* = 0.16). Table 4 provides an overview over means and standard deviations both when the 69 suspicious respondents are excluded and included.

**Table 4:** Hireability judgments per condition for Study 3.

		***WHPP Focus***	
		**Organizational**	**Individual**
**SUSPICIOUS RESPONDENTS EXCLUDED (*****n*** = **169)**			
*Candidate Weight Status*	Non-overweight	5.99 (1.00)^a^	6.00 (0.83)^a^
	Overweight	5.91 (1.10)^a^	5.69 (1.12)^b^
**SUSPICIOUS RESPONDENTS INCLUDED (*****n*** = **238)**			
*Candidate Weight Status*	Non-overweight	5.89 (1.05)^a^	5.91 (0.86)^a^
	Overweight	5.84 (1.17)^a^	5.67 (1.08)^b^

*Within columns, subscripts that share a letter do not differ significantly*.

#### Hypotheses testing: weight bias internalization

Hypotheses 5a and 5b stated that the WHPP emphasizing individual responsibility would increase weight bias internalization, and that this effect would be more pronounced for people with a high BMI. To test this, we effect coded WHPP Focus (−1 = individual responsibility, 1 = organization responsibility), standardized BMI and from this calculated WHPP × BMI interaction term. These variables were regressed on weight bias internalization. This rendered a significant model (no outliers were detected), *F*_(2, 229)_ = 32.46, *p* < 0.001, *R*^2^ = 0.22 and a main effect of BMI, β = 0.70, *t* = 7.93, *p* < 0.001, Cohen's *d* = 1.22, showing that respondents with a higher BMI reported more weight bias internalization than respondents with a lower BMI. There was no effect of WHPP Focus (β = −0.04, *p* = 0.49, Cohen's *d* = 0.10) nor a significant WHPP × BMI interaction, β = 0.01, *p* = 0.82, Cohen's *d* = 0.03). As people's self-perception of whether they are with overweight or obesity may depend more on whether their BMI falls into the “overweight” or “obesity” categories than on their exact BMI value, we also conducted an analysis with BMI as a categorical variable, making categories based on overweight (BMI ≥ 25 kg/m^2^) or obesity (BMI ≥ 30 kg/m^2^). However, there were no interactions between WHPP Focus and respondents' BMI. Power analysis for multiple regression showed a power of 0.99 to find a medium effect size of f^2^ = 0.15 using G^*^Power (Faul et al., 2007).

##### Conclusions hypothesis 5

Hypotheses 5a and 5b that WHPP Focus affects weight bias internalization, and that this effect is stronger for employees with a higher BMI were not supported. Rather, higher BMI generally was associated with greater weight bias internalization amongst employees.

#### Exploratory analyses: targets' perspective

Next to our preregistered hypotheses, we also performed a number of exploratory analyses in order to gain more insights into potentially different effects of WHPP on employees with or without overweight. More specifically, we tested the effects of WHPP Focus and respondents' BMI on our two measures of controllability of overweight (the slider measure and BAOP). We first tested this regarding BMI as a continuum and then regarding with BMI as weight status categories (contrasting “with overweight” vs. “without overweight” and contrasting “with obesity” vs. “without obesity”).

##### BMI as continuous variable

Regressing the slider measure of perceived controllability of overweight on WHPP Focus, BMI and their interaction term rendered only a marginal main effect of BMI, β = −0.11, *t* = −1.73, *p* = 0.086, Cohen's *d* = 0.23. Regressing BAOP on WHPP Focus, BMI and their interaction rendered a significant main effect of BMI, β = −0.19, *t* = −2.87, *p* < 0.005, Cohen's *d* = 0.38. This shows that respondents with a relative high BMI perceived weight as being *less* controllable than respondents with a relative low BMI. In both regressions, no effect of WHPP Focus or a BMI by WHPP Focus interaction was evident. Power analysis for multiple regression showed a power of 0.99 to find a medium effect size of f^2^ = 0.15 using G^*^Power (Faul et al., 2007).

##### BMI as categorical variable

We tested whether controllability attributions were determined by respondents' BMI *category* rather than their exact BMI. We indeed found support for this when comparing respondents with and without obesity. Specifically, we tested the influence of WHPP Focus (Responsibility: individual vs. organizational) and respondents' BMI (BMI Category: without obesity vs. with obesity) on controllability perceptions by means of a multivariate ANOVA with the slider measure and BAOP as dependent variables. For both indicators of perceived controllability of weight, no main effects of WHPP Focus and BMI Category were found, but significant interactions between the independent variables were revealed for the slider measure, *F*_(2, 232)_ = 6.05, *p* = 0.01, Cohen's *d* = 0.32, and for BAOP, *F*_(2, 232)_ = 3.94, *p* = 0.048, Cohen's *d* = 0.26. Table 6 shows means and standard deviations. Tests for simple main effects for both measures of perceived controllability of weight revealed that respondents with and without obesity did not differ in perceived controllability of weight when the WHPP emphasized organizational responsibility, both *F*'s_(1, 227)_ < 2.3, both *p*'s > 0.13. By contrast, respondents with obesity perceived weight to be significantly less controllable than respondents without obesity when the WHPP emphasized individual responsibility [Slider measure: *F*_(1, 227)_ = 4.09, *p* = 0.04, Cohen's *d* = 0.26; BAOP: *F*_(1, 227)_ = 5.84, *p* = 0.016, Cohen's *d* = 0.32]. Power analyses for this analysis showed a power of 0.99 to detect a medium effect size of f = 0.25 using G^*^Power (Faul et al., 2007).

##### Conclusions exploratory analyses

The exploratory results suggest that people with obesity feel that overweight is less controllable than people without obesity, but that this is only the case when confronted with a WHPP that emphasizes individual responsibility (Table 5). So, one the one hand, our investigation shows that WHPP emphasizing individual responsibility causes *all* employees to ascribe responsibility for health to the individual employee (as the manipulation checks presented earlier suggest). On the other hand, employees with obesity perceive weight to be significantly *less* controllable in such situations. We will elaborate on the significance of this finding in more detail in the discussion section.

**Table 5:** Correlations between control variables, independent and dependent variables, Study 3.

		**1**	**2**	**3**	**4**	**5**	**6**	**7**	**8**	**9**	**10**
Control variables	1. Gender (1 = male, 2 = female)	1.00								
	2. Age	0.04	1.00							
	3. Education	−0.01	0.00	1.00						
	4. Work in HR	0.11	0.02	0.11	1.00					
	5. Hiring Experience	−0.03	0.22*	0.23**	0.52**	1.00				
	6. BMI	0.03	0.08	−0.20*	−0.03	0.06	1.00			
IVs	7. WHPP focus^a^	−0.07	−0.06	0.04	0.01	0.08	0.09	1.00		
DVs	8. Weight discrimination	−0.12	−0.09	0.16*	−0.02	0.05	−0.04	0.13*	1.00	
	9. Weight bias internalization	0.20**	−0.07	−0.05	0.11	0.01	0.47**	0.07	0.01	1.00
	10. Controllability (slider)^b^	−0.17**	−0.02	−0.04	−0.10	−0.07	−0.11	−0.02	0.11	−0.15*	1.00
	11. Controllability (BAOP)^b^	−0.12^+^	−0.01	−0.08	−0.18**	−0.20**	−0.18*	0.03	0.18*	−0.19**	0.58**

a *1 = individual responsibility (N = 121), −1 = organizational responsibility (N = 117)*.

b *Higher values indicate greater perceived controllability*,

+ *p = 0.065*,

* *p < 0.01*,

** *p < 0.001*.

**Table 6:** Perceived controllability of weight (slider measure and BAOP) as a function of WHPP Focus and BMI category (without overweight vs. with overweight), Study 3.

		***WHPP Focus***	
		**Organizational**	**Individual**
**SLIDER MEASURE**			
*Candidate Weight Status*	Without overweight	70.56 (20.72)^a^	76.81 (19.85)^a^
	With overweight	77.68 (17.93)^a^	66.56 (25.16)^b^
**BAOP**			
*Candidate Weight Status*	Without overweight	4.41 (0.80)^a^	4.62 (0.82)^a^
	With overweight	4.51 (0.91)^a^	4.17 (0.90)^b^

*Within columns, subscripts that share a letter do not differ significantly*.

## General discussion

The findings in this paper show that WHPP emphasizing individual responsibility induce weight stigma and discrimination in the workplace. We reasoned that workplace health promotion, especially when the program emphasizes individual responsibility, would contribute to the perception that weight is controllable, and that this would evoke weight stigma and weight-based discrimination. Our data shows that this is not the complete story. We found that the mere presence of a WHPP leads to stronger beliefs that weight is controllable (Studies 1 and 2). However, WHPP focus (i.e., individual or organization responsibility) did not affect beliefs about the controllability of weight but did affect weight stigma and weight-based discrimination. Thus, whilst weight stigma and discrimination were not the result of a belief that weight is controllable, a focus on individual responsibility within a WHPP did lead to increased weight stigma and discrimination.

Thus, the increase in weight stigma and discrimination observed in our studies was not caused by changes in controllability beliefs. This aligns with decision stage models of attribution (e.g., Mantler et al., 2003). These models entail that attributions of controllability, ascriptions of responsibility, and target blame are hierarchical constructs that prompt social observers to infer from the presence of the higher order construct that the lower order constructs are present too (Mantler et al., 2003). In other words, when social observers blame the target, they will assume responsibility for and controllability over the outcome. Likewise, when social observers ascribe responsibility for an outcome such as overweight, they will infer that the other person had control over the outcome. Hence, blaming and responsibility ascriptions may not always be the result of controllability perceptions but may actually cause them. This may be the reason why, in the current investigation, WHPP focus (which are responsibility ascriptions) did not affect stigma and discrimination through controllability perceptions. This also aligns with the notion that moral evaluations based on obesity are often implicit (Hoverd and Sibley, 2007) and thus need not to arise from controllability attributions. Indeed, a general feeling of dislike was also reported by Pescud et al. (2015), who found that employers' views of “unhealthy workers” involved perceptions such as “unpleasant company.”

Our findings contribute to both existing literature and practice in three important ways. First, the insights offered in this research contribute to what we know about WHPP. Research on WHPPs has, so far, only focused on health-related outcomes such as sick leave, physical activity, and workplace wellness (Anderson et al., 2009; Odeen et al., 2012; Osilla et al., 2012; Malik et al., 2014). These systematic reviews concerning the impact of WHPP on employee health in several domains have revealed sobering conclusions. We have suggested that this is at least partly due to a general failure to consider the complex interplay between individuals and their social environments—such as the organizations they work for—when employee health is concerned. The current investigation is the first to focus on the negative side-effects of WHPP in terms of stigmatization and discrimination of employees with overweight and obesity. In addition, we studied the effect of framing WHPP differently and found that weight-stigma and weight-based discrimination can be prevented by WHPP emphasizing organizational rather than individual responsibility.

Second, our findings contribute to existing insights about weight stigma. Prior research already showed that people with overweight and obesity are stigmatized and face discrimination in the workplace based on weight (e.g., O'Brien et al., 2008; Flint et al., 2016). However, our findings extend existing evidence by experimentally examining the effects of WHPP and their emphasis of either individual or organizational responsibility. This research is both novel and timely given the rapid rise in WHPP that aim to support employee health, many of which aim to “support employees in weight management.” The workplace represents a setting where weight stigma and discrimination is reported, and there is a need to reduce these experiences. Thus, when designing WHPP, organizations should ensure that they support employee health and avoid potential counterproductive effects such as weight stigma and discrimination as observed in our studies.

Third, our finding contribute to the literature on the effect of changeability beliefs concerning weight (the “stigma asymmetry model,” see Burnette et al., 2017; Hoyt et al., 2017), also mentioned in the introduction. This literature shows that the belief that obesity is an unchangeable disease has opposite effects on weight stigma through different paths. On the one hand, it reduces blame, but, on the other hand, it fosters the view of people with obesity having an unchangeable essence, thus fostering an essentialist view. Our data is in line with this model and suggest stigma asymmetry extends beyond the “obesity as a disease” issue and into the domain of WHPP. After all, we studied not only the focus of WHPP but also its mere presence. When a WHPP is present, this suggests that obesity is changeable (either by individual themselves or by their organizational environment). In Study 2, the mere presence of the WHPP did not affect weight stigma. This could be due to the fact that the WHPP on the one hand (or: for some people) increases blame, increasing weight stigma, and on the other hand (or: for other people), reduced an essentialist view of obesity, decreasing weight stigma. Our data further suggest that, only when changeability is connected to controllability by an *individual* rather than the environment, it increases weight stigma.

### The target's perspective

With regard to the targets' perspective, although our findings in Study 2 suggest that the presence of a WHPP emphasizing individual responsibility increases the weight bias of people with a relative high BMI, Study 3 did not support the idea that it increased weight bias internalization in employees with overweight and obesity. However, the WHPP's emphasis on individual responsibility did appear to decrease the belief that weight is controllable particularly in people with obesity. On first sight, this may seem like a manifestation of resistance against the message that individuals are responsible for their own weight, a message that is arguably threatening for people with obesity (e.g., Dillard and Shen, 2005). However, our data does not support this interpretation. As became clear from the manipulation checks in Study 3, people with obesity were convinced by the WHPP emphasizing individual responsibility that employees were to be held responsible. Thus, when involved with a WHPP emphasizing individual responsibility, employees with obesity respond with a disturbing combination of feeling personally responsible for their weight, whilst perceiving little controllability of weight.

From a motivational perspective, people with obesity are thus likely to be caught in a Catch-22 like situation, which can result in maladaptive responses. After all, insights from learned helplessness theory show that, when people feel responsible for uncontrollable events, this harms their self-esteem (Abramson et al., 1978; Alloy et al., 1988; Pierce and Wardle, 1997) and potentially results in diet-breaking behavior and weight gain (Ogden and Wardle, 1990; Townend, 2009). Indeed, those targeted by moralized views of others often respond maladaptively. For instance, Mulder et al. (2015) showed that when confronted with moralizing health messages, participants with overweight ate more unhealthy snacks than when they were confronted with counter-moralizing health messages. Future research should thus explore the impact of public views of the morality of obesity on motivation for dieting and exercise in people with overweight and obesity.

In sum, the current research suggests that emphasizing individuals' responsibility for employee health in WHPP leads to a moral burden compounded by employees with obesity feeling that they are unable to influence the outcome. This is likely to have a demotivating effect. Thus, it could very well be that a WHPP, to the extent that it emphasizes individual responsibility, fails to evoke healthy behavior amongst employees with overweight and obesity. Further, WHPP emphasizing individual responsibility also lead to employees with overweight and obesity being targeted by stigma and discrimination. Both are associated with several unwarranted outcomes, such as decreased mental and physical health (Pascoe and Smart Richman, 2009; Puhl and Suh, 2015), increased healthcare costs (Osumili et al., 2016), and underperformance (Glover et al., 2017). Thus, if not implemented carefully, WHPP might have negative rather than the expected positive effects.

### Strengths, limitations, and future research

Due to the experimental designs employed in Studies 2 and 3, the research presented here allows for causal inferences regarding the effects of WHPP presence and focus on employees' controllability perceptions, weight stigma, and weight-based discrimination. Further, both Studies 1 and 3 included varied samples of US citizens, and a diverse range of BMI. Study 3 in particular involved a great number of people who reported making hiring decisions in their work context. An additional strength of particularly Study 3 was that the hypotheses and analytical strategy were preregistered. This approach safeguards the confirmatory (rather than exploratory) nature of our data analysis and offers a transparent approach to *post-hoc* analyses and interpretations (Lindsay et al., 2016; Nosek and Lindsay, 2018). Together, this makes us confident that our results are credible and generalizable.

A limitation of our research is that we exclusively focused on WHPP effects on weight stigma and weight-based discrimination, thereby excluding a range of other health-related behaviors and outcomes. The question thus remains whether our results would also generalize to people with, for instance, burn-out, cancer, or chronic diseases. Based on extensive research into the role of controllability and responsibility attributions on blame (e.g., Weiner et al., 1988; Weiner, 1995; Mantler et al., 2003), emphasizing individual responsibility for health may affect stigma toward other health-related behaviors and outcomes in the same way. Recent research shows that health moralization—which is strongly associated with responsibility—prompts people who live healthily to stigmatize and discriminate against others who live less healthy (Täuber, 2018). This effect was also evident for non-weight related health outcomes such as smoking, an unhealthy lifestyle more generally, and even for being ill. Nevertheless, overweight is more strongly associated with lifestyle than many (other) diseases such as cancer or burnout. Therefore, WHPP emphasizing individual responsibility may affect weight stigma more than stigma based on non-lifestyle related diseases. The results of Study 1 indeed support this notion as the WHPP presence only predicted controllability perceptions with regard to overweight and not with regard to cancer or burnout. More research is needed to test the effects of WHPP presence and focus on stigma based on diseases other than overweight.

Further, we manipulated WHPP focus rather than studying the focus of existing WHPP as they are implemented in organizations. The advantage of this is that we could establish causal relations and draw robust conclusion about the effects of a WHPP's individual vs. organizational responsibility focus. We assumed that most WHPP are implemented with a focus on individual responsibility. This was based on the notion that these programs are often employer-driven, and employers typically see health as employees' responsibility (Meershoek et al., 2010; van Berkel et al., 2014), as well as on the identified shift in focus from occupational health protection (responsibility of employers) to occupational health promotion (responsibility of employees; Macdonald and Sanati, 2010). However, the *extent* to which actual WHPP reflect this individual focus, and how this extent contributes to weight bias and discrimination, is a topic for further research.

Finally, to highlight the target's perspective, we focused on the influence of BMI. However, a valuable extension of this would be to take into account self-perceived weight rather than BMI. Prior research (Major et al., 2014) points out that people who do not perceive themselves as overweight feel less threatened by weight-stigmatizing messages, even when they are objectively overweight. Thus, WHPP focusing on individual responsibility might have less negative effects on employees with overweight or obesity but do not perceive themselves as such, and a more negative effects on employees without overweight or obesity but do perceive themselves as such. This is a notion that future research might examine.

## Conclusion

Our findings suggest that the implementation of WHPP affects employees' perceptions of the controllability of weight, and the WHPP focus on individual (rather than organizational) responsibility leads to weight stigma and weight-based discrimination. These consequences have to be considered severe, particularly in light of the prevalence of employees with overweight and obesity (e.g., World Health Organisation, 2010), and the ever-increasing number of WHPP aiming to promote health at the workplace (e.g., Chen et al., 2015). Our research thus suggests that WHPP might be less beneficial for employees than commonly expected, especially when they emphasize individual responsibility for health. Specifically, our results demonstrate that a clear communication of *organizational* rather than individual responsibility for health might interrupt the automatic association of controllability with responsibility and ultimately blame (Weiner et al., 1988; Weiner, 1995, 2000; Crandall et al., 2001). In addition, such organizational responsibility attribution may induce the right motivation of those targeted to change their behavior. This notion is based on insights showing that using non-moral language is more motivating than using moralized language, which holds for diverse topics such as climate change (Täuber et al., 2015), poverty reduction (Täuber and van Zomeren, 2012), immigration policy (Täuber and van Zomeren, 2013), and obesity (Mulder et al., 2015).

This is a valuable insight for practitioners, particularly for human resource management concerned with the design and implementation of WHPP. Our research suggests that to attenuate weight-based stigmatization and weight-based discrimination, WHPP should be designed and communicated in ways that emphasize the responsibility of the organization rather than of the individual employee. This can be done, for instance, by creating healthy organizational environments where mostly healthy food is offered in the canteen (rather than simply informing employees about what healthy eating is), by providing offices with standing desks, or by giving the stairs a more prominent placing than the elevator. In addition, in communication about the WHPP it is important that the focus should lie on the responsibility of the organization rather than the individual employee (e.g., communicate that the healthy food offering in the canteen is meant to make it easier for employees to eat healthier rather than to encourage employees to make healthy choices). Based on our findings, we recommend HR managers and other professionals involved in designing and implementing WHPP to critically review their policies regarding who is held responsible for employee health—even if this is implied rather than explicitly formulated in the policy.

## Author contributions

ST and LM contributed equally to this paper and designed, carried out and performed the statistical analyses of study 2. LM designed, carried out and analyzed study 1 and performed the statistical analyses of study 3. ST, LM, and SF designed and carried out study 3. ST wrote the first draft of the manuscript. All authors contributed to writing the manuscript and have read and approved the submitted version.

### Conflict of interest statement

The authors declare that the research was conducted in the absence of any commercial or financial relationships that could be construed as a potential conflict of interest.

## Footnotes

^1^In a pilot study (*N* = 56) designed to create the materials for this study, a paired sample t-test revealed no differences in hireability ratings between these two CV's, *t*_(55)_ = 1.05, *p* = 0.30.

## Supplementary Material

The Supplementary Material for this article contains the appendix and can be found online at: https://www.frontiersin.org/articles/10.3389/fpsyg.2018.02206/full#supplementary-material
