# Supplementary material for: The Impact of Workplace Health Promotion Programs Emphasizing Individual Responsibility on Weight Stigma and Discrimination
Source: Front Psychol. 2018 Nov 19;9:2206. doi: 10.3389/fpsyg.2018.02206 (PMC6253158; doi:10.3389/fpsyg.2018.02206)
Supplement: Supplementary file 1 [file Data_Sheet_1.docx]

**Appendix**

**Study 2**

**Manipulation**

| **WHPP absent** | **WHPP present** | | |
| --- | --- | --- | --- |
|  | **no information** | **responsibility organization** | **responsibility individual** |
| The university finds it important that her employees and students are healthy: that they have a good condition and no overweight. | | | |
|  | Therefore, the university is planning to take several measures to promote the health of our employees and students. | | |
|  | The buildings will be slightly adapted so that the stairs will get a more prominent place than the elevator. | | |
|  |  | In this way, taking the stairs become the more “logical option” and people automatically will be more inclined to take the stairs instead of the elevator | In this way, people will be motivated to take the stairs instead of the elevator |
|  | In canteens, we will make sure there is an extensive offer of healthy food. | | |
|  |  | This makes it easier for students and employees to choose for healthy food. As such, students and employees shall be to a lesser extent be lead into temptation to choose unhealthy snacks. | This makes it possible for students and employees to choose for healthy food. They will have the choice not to be tempted by unhealthy snacks. |
|  | Also, there will be more sport facilities for employees and students. Our aspiration is to make sure that, in each building where employees and students come, there will be at least one sports area in which people can engage in, for example, fitness. | | |
|  |  | This is to make it easier for employees and students to engage in sport in between work and study activities. | This is to stimulate employees and students to engage in sport in between work and study activities. |
|  |  | In this way, the de university takes her responsibility to promote the health of employees and students. | In this way, the university appeals to the employees and students to take their responsibility in promoting their own health. |

**Measures**

*Perceived overweight*

I think I'm overweight.

I think I'm fat.

I am satisfied with my weight.

I am currently trying to lose weight.

I have tried to lose weight in the past.

I would like to be thinner.

At the moment I weigh more than my ideal weight.

*Controllability attributions*

People have little influence on their weight. (reverse coded)

Overweight is something that people cannot change themselves. (reverse coded)

*Weight stigma (fat phobia)*

The following pictures were shown (anonymized for the purpose of this paper. In Studies 2 and 3 the pictures were shown to respondents without anonymization):


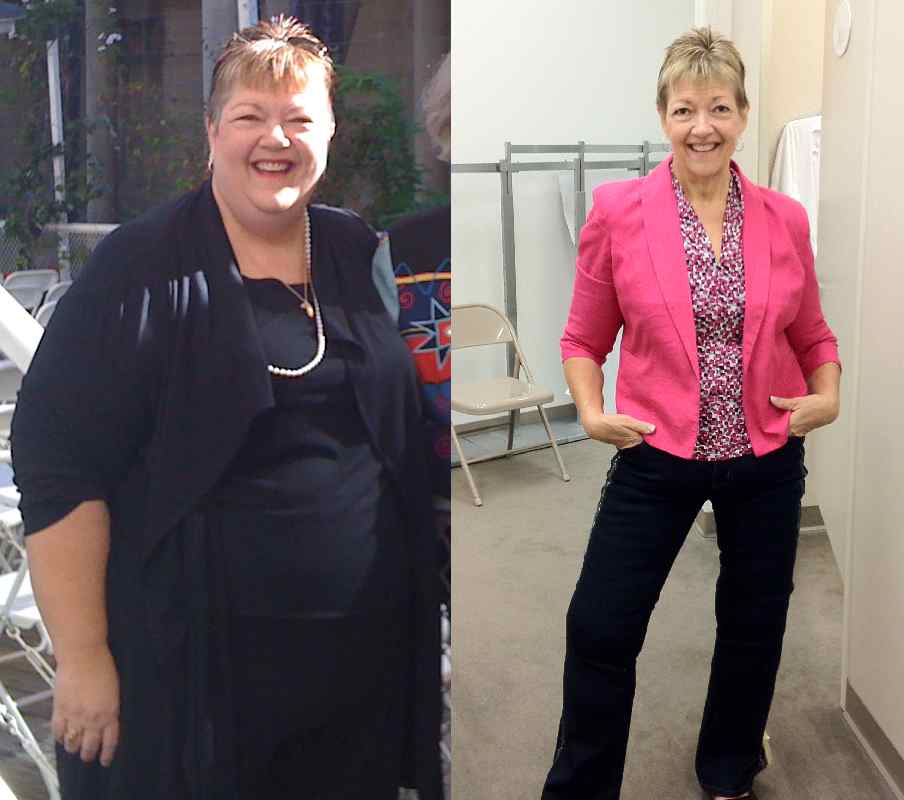


Please indicate to what extent you think the following traits apply more to the woman on the left side, more the woman on the right side or to both women equally

industrious, has willpower, attractive, slow, persevering, active, weak, self-indulgent, food-loving, insecure, has a high self-esteem, well-shapely, overeats, has good self-control

**Additional analyses Study 2**

In a pilot test we presented both pictures to 20 respondents and asked them to rate them on various attributes on a 7-point Likert scale. Also, respondents also estimated the age of the two women in the pictures. The scales and results are presented in the Table below.

| *Attribute* | *Scale* | | *M_woman with overweight_* | *M_woman without overweight_* | *t* (19) | *p* |
| --- | --- | --- | --- | --- | --- | --- |
| Weight | | 1 = unhealthy weight, 7 = healthy weight | 1.55 | 5.25 | -8.87 | <.001 |
| Posture | | 1 = thin,  7 = fat | 6.40 | 3.45 | 10.69 | <.001 |
| Attractiveness | | 1 = unattractive,  7 = attractive | 1.90 | 2.75 | -3.22 | .005 |
| Dressing style | | 1 = well dressed,  7 = badly dressed | 3.70 | 3.10 | 1.64 | .12 |
| Competence | | 1 = incompetent,  7 = competent | 4.4 | 4,6 | -0.54 | .60 |
| Friendliness | | 1 = unfriendly,  7 = friendly | 5.75 | 5.20 | 2.60 | .02 |
| Age | | Answer in years | 50.9 | 54.9 | -2.20 | .04 |

**Study 3**

**Actions in WHPP as used in the manipulation in Study 3**

| **WHPP focus: Individual** | **WHPP focus: Organizational** |
| --- | --- |
| In the canteen, encourage employee to eat healthily by special promotions and posters/leaflets encouraging them to make healthy choices. | In the canteen, all unhealthy food will be removed and replaced by healthy food only. |
| In the hallway, encourage employees to take the stairs rather than the elevator by signs (“Take the stairs!”). | The hall will be redesigned so that the stairs appear more prominent and the elevator will be relocated in a less prominent place. Employees will then automatically be more inclined to take the stairs without having to think about it. |
| Employees will be offered to engage in a health check. This health check will give an indication of the employee's health status and will suggest action plans to improve it. For example, getting support from a dietician, engaging in sports, etc. Following up on these actions is at the employee’s own cost. | Employees will be offered to engage in a health check. This health check will give an indication of the employee's health status and will suggest action plans to improve it. For example, getting support from a dietician, engaging in sports, etc. Sturdation will cover the costs of following up on these action plans. . |
| Install software on computers that remind employees to take a different posture, take a break, stand up, or move around | Offices will be refurnished with “standing desks”, to enable employees to work whilst standing rather than sitting. |

**Measures**

*Weight Bias Internalization (WBI; Pearl & Puhl 2014)*

Because of my weight, I feel that I am just as competent as anyone. (reverse)

I am less attractive than most other people because of my weight.

I feel anxious about my weight because of what people might think of me.

I wish I could drastically change my weight.

Whenever I think about my weight, I feel depressed.

I hate myself for my weight.

My weight is a major way that I judge my value as a person.

I don't feel that I deserve to have a really fulfilling social life, because of my weight.

I am OK being the weight I am. (reverse)

Because of my weight, I don't feel like my true self.

Because of my weight, I don't understand how anyone attractive would want to date me.

*Beliefs About Obese Persons Scale (BAOP; Allison, Basile, & Yuker, 1991)*

Obesity often occurs when eating is used as a form of compensation for lack of love or attention.

In many cases, obesity is the result of a biological disorder. (reverse)

Obesity is usually caused by overeating.

Most obese people cause their problem by not getting enough exercise.

Most obese people eat more than nonobese people.

The majority of obese people have poor eating habits that lead to their obesity.

Obesity is rarely caused by a lack of willpower. (reverse)

People can be addicted to food, just as others are addicted to drugs, and these people usually become obese.

*Hireability Judgments*

How competent do you feel the candidate is for this job? (from 1 = extremely incompetent to 7 = extremely competent)

How likely is it that the candidate has the necessary skills for this job? (from 1 = extremely unlikely to 7 = extremely likely)

How qualified do you think the candidate is for this job? (from 1 = extremely unqualified to 7 = extremely qualified)

How likely would you be to hire the applicant for the senior policy advisor job? (from 1 = extremely unlikely to 7 = extremely likely).

**Additional analyses Study 3**

We also tested the influence of WHPP and BMI on a related exploratory dependent variable, namely the feeling of being valued and respected by colleagues and the organization. The scale was composed of four items asking respondents’ agreement with the statements “If I worked at Sturdation, I would feel... respected by colleagues,...respected by the managing board,...valued for who I am, and...valued for my competencies rather than my appearance (α=.91). This rendered a significant model, *F*(2,229) = 6.99, *p*=.001, *R^2^* = .05. WHPP Focus exerted a main effect on this measure, β = -.22, *t* = 3.38, *p*=.001, showing that respondents felt less respected and valued when the WHPP emphasized individual, as opposed to organizational, responsibility. This effect was not moderated by respondents’ BMI (β = -.04, *p* = .486), and no main effect of BMI was evident (β = .46, *t* = 7.93, *p* < .001). This suggests that the detrimental effects of WHPP emphasizing individual responsibility apply to employees irrespective of their weight. In other words, WHPP stressing individual responsibility for health decrease feelings of being respected and valued for everyone at the workplace.
